# Applying surface-based hippocampal morphometry to study APOE-E4 allele dose effects in cognitively unimpaired subjects

**DOI:** 10.1016/j.nicl.2019.101744

**Published:** 2019-03-04

**Authors:** Qunxi Dong, Wen Zhang, Jianfeng Wu, Bolun Li, Emily H. Schron, Travis McMahon, Jie Shi, Boris A. Gutman, Kewei Chen, Leslie C. Baxter, Paul M. Thompson, Eric M. Reiman, Richard J. Caselli, Yalin Wang

**Affiliations:** a School of Computing, Informatics, and Decision Systems Engineering, Arizona State University, Tempe, AZ, USA; b Wellesley College, Wellesley, MA, USA; c Armour College of Engineering, Illinois Institute of Technology, Chicago, IL, USA; d Banner Alzheimer's Institute, Phoenix, AZ, USA; e Human Brain Imaging Laboratory, Barrow Neurological Institute, Phoenix, AZ, USA; f Imaging Genetics Center, Institute for Neuroimaging and Informatics, University of Southern California, Los Angeles, CA, USA; g Department of Neurology, Mayo Clinic Arizona, Scottsdale, AZ, USA

**Keywords:** APOE-e4, Hippocampal morphometry, Magnetic resonance imaging (MRI), Alzheimer's disease, Cognitively unimpaired

## Abstract

Apolipoprotein E (APOE) e4 is the major genetic risk factor for late-onset Alzheimer's disease (AD). The dose-dependent impact of this allele on hippocampal volumes has been documented, but its influence on general hippocampal morphology in cognitively unimpaired individuals is still elusive. Capitalizing on the study of a large number of cognitively unimpaired late middle aged and older adults with two, one and no APOE-e4 alleles, the current study aims to characterize the ability of our automated surface-based hippocampal morphometry algorithm to distinguish between these three levels of genetic risk for AD and demonstrate its superiority to a commonly used hippocampal volume measurement. We examined the APOE-e4 dose effect on cross-sectional hippocampal morphology analysis in a magnetic resonance imaging (MRI) database of 117 cognitively unimpaired subjects aged between 50 and 85 years (mean = 57.4, SD = 6.3), including 36 heterozygotes (e3/e4), 37 homozygotes (e4/e4) and 44 non-carriers (e3/e3). The proposed automated framework includes hippocampal surface segmentation and reconstruction, higher-order hippocampal surface correspondence computation, and hippocampal surface deformation analysis with multivariate statistics. In our experiments, the surface-based method identified APOE-e4 dose effects on the left hippocampal morphology. Compared to the widely-used hippocampal volume measure, our hippocampal morphometry statistics showed greater statistical power by distinguishing cognitively unimpaired subjects with two, one, and no APOE-e4 alleles. Our findings mirrored previous studies showing that APOE-e4 has a dose effect on the acceleration of brain structure deformities. The results indicated that the proposed surface-based hippocampal morphometry measure is a potential preclinical AD imaging biomarker for cognitively unimpaired individuals.

## Highlights

• Applied surface-based hippocampal morphometry on cognitively unimpaired subjects.
• Our study identified APOE-e4 dose effects on cognitively unimpaired subjects.
• Surface-based hippocampal morphometry outperformed the hippocampal volume measure.
• Surface-based hippocampal morphometry may be a potential preclinical AD biomarker.

## 1 Introduction

Alzheimer's disease (AD), a progressive form of dementia that interferes with memory, thinking, and behavior, is one of the main threats to the life quality of the elderly. Cognition decline especially memory loss is the clinical hallmark of AD onset, resulting from an irreversible neurodegenerative progress. For therapy to be successful, timing may be critical, and current trial trends have emphasized intervention at the earliest possible stage, including pre-symptomatic. However, it is challenging to determine obvious AD in the pre-symptomatic stage (Hyman, 2011). Discovery that a common gene, the apolipoprotein E (APOE) e4 allele, is the major genetic risk factor for late-onset AD (Corder et al., 1993; Saunders et al., 1993), has made it possible to study large numbers of genetically at-risk individuals before the onset of symptomatic memory impairment and has led to the concept of the *preclinical stage of AD* (Sperling et al., 2011), a concept validated in autopsy studies of non-demented elderly subjects with neuropathological evidence of AD at autopsy (Dickson et al., 1992; Gouras et al., 1997; Bennett et al., 2009; Kok et al., 2009; Caselli et al., 2010), magnetic resonance imaging (MRI) studies of infants at differential genetic risk (Dean 3rd et al., 2014; Knickmeyer et al., 2014), MRI studies of whole brain atrophy rates (Chen et al., 2007), fluorodeoxyglucose positron emission tomography (FDG-PET) studies of APOE-e4 carriers that have revealed AD-like patterns of reduced CMR glucose (Reiman et al., 1996; Reiman et al., 2005), amyloid ligand binding studies using Pittsburgh Imaging Compound B (PiB) that show evidence of cerebral amyloidosis in APOE-e4 carriers (Reiman et al., 2009), cerebrospinal fluid (CSF) levels of beta amyloid that begin to fall, suggesting the onset of AD, in the early 50's in e4 carriers (Morris et al., 2010), and neuropsychological studies showing the accelerated decline of memory scores in a gene-dose pattern in APOE-e4 carriers beginning between age 55 and 60 (Caselli et al., 2009) that is further accelerated in APOE-e4 homozygotes by cerebrovascular risk factors (Chen et al., 2007; Caselli et al., 2011).

In AD research, structural MRI-based measures include whole-brain (Fox et al., 1999; Chen et al., 2007; Josephs et al., 2008), entorhinal cortex (Cardenas et al., 2011), hippocampus (Reiman et al., 1998; Jack Jr. et al., 2003; Thompson et al., 2004a; den Heijer et al., 2010; Wolz et al., 2010), and temporal lobe volumes (Hua et al., 2010), as well as ventricular enlargement (Jack Jr. et al., 2003; Thompson et al., 2004a; Wang et al., 2011). These measures correlate closely with differences and changes in cognitive performance, supporting their validity as markers of disease progression. In particular, the hippocampus is a primary target region in both cross-sectional and longitudinal structural MRI analysis of AD progress (de Leon et al., 1989; Soininen et al., 1995; Reiman et al., 1998; Wang et al., 2003; Thompson et al., 2004a; Qiu et al., 2009; Shen et al., 2009; Apostolova et al., 2010; Crivello et al., 2010; O'Dwyer et al., 2012; Kerchner et al., 2014), and studies show that the presence of more APOE-e4 alleles results in increased hippocampal atrophies on AD (Filippini et al., 2009; Hostage et al., 2013; Kerchner et al., 2014; Chen et al., 2016; Saeed et al., 2018), mild cognitive impairment (MCI) (Hostage et al., 2013; Chen et al., 2016) and non-demented subjects (pooled MCI and cognitively impaired subjects) (Shi et al., 2014; Li et al., 2016).

Evaluating the genetic influence of APOE-e4 on hippocampal morphology before the onset stage of AD may enrich our understanding of the involvement of this allele in AD pathology, and have implications for prevention strategies. In an early study involving 11 cognitively normal APOE-e4 homozygotes (HM) and 22 APOE-e4 non-carriers (NC) with a reported family history of AD who were matched for sex, age, and level of education, Rieman et al. (1998) reported that the HM showed nonsignificant trends for smaller left and right hippocampal volumes. By studying the dose effect of APOE-e4 on hippocampal volume loss in a large MRI database of cognitively unimpaired subjects, a series of studies (Lemaitre et al., 2005; Crivello et al., 2010) found significant hippocampal atrophies in HM compared to heterozygotes (HT) and NC but observed no significant morphometric differences between HT and NC/HM. Another popular research strategy pooled HM and HT into a single APOE-e4 carrier category. Significantly smaller hippocampal volumes were observed in APOE-e4 carriers than in NC (Reiter et al., 2017), but the hippocampal volumes of HT were not significantly different from NC. Similarly, voxel-wise techniques did not report meaningful findings about APOE-e4 dose effects (Matura et al., 2014; Gonneaud et al., 2016). Recently, Cacciaglia et al. (2018) found APOE-e4 additive grey matter volume reductions in the right hippocampus, caudate, precentral gyrus, and cerebellar.

Although the majority of existing studies used hippocampal volumes (Reiman et al., 1998; Lemaitre et al., 2005; Crivello et al., 2010; Reiter et al., 2017; Cacciaglia et al., 2018), recent research (Styner et al., 2004; Thompson et al., 2004a; Morra et al., 2009; Qiu et al., 2009; Shen et al., 2009; Apostolova et al., 2010; Costafreda et al., 2011; Younes et al., 2014) has demonstrated that surface-based subregional structure analysis may offer advantages over volume measures. However, the dose-dependent impact of the APOE-e4 allele measured by a surface-based hippocampal morphometry system on cognitively unimpaired individuals is still elusive.

In our previous studies (Wang et al., 2011; Shi et al., 2014), we proposed a novel multivariate measure of hippocampal morphometry to analyze the hippocampal surface deformations related to APOE-e4 dose effects and validated it on the Alzheimer's Disease Neuroimaging Initiative (ADNI) dataset (adni.loni.usc.edu) with known APOE genotypes (167 subjects with AD, 354 subjects with MCI, and 204 cognitively unimpaired subjects). The proposed surface multivariate morphometry statistics (MMS) consist of multivariate tensor-based morphometry (mTBM) (Leporé et al., 2008; Wang et al., 2010) and radial distance (distances from the medial core to each surface point) (Styner et al., 2004; Thompson et al., 2004a). With surface MMS, our experimental results have shown that APOE-e4 exerts dose effects on the left hippocampus (LH) of non-demented individuals (i.e., atrophies of LH_HM_ > atrophies of LH_HT_ > atrophies of LH_NC_) (Shi et al., 2014; Li et al., 2016). Meanwhile, the hippocampal morphometric measures have been verified in our previous AD-related neuroimaging research, showing stronger statistical power than volume-based analysis in capturing subtle structural alterations (Wang et al., 2011; Shi et al., 2014; Zhang et al., 2016).

In the present study, we hypothesized that our unique automated hippocampal morphometry system (Shi et al., 2014) may help reveal the dose effects of APOE-e4 on the hippocampal morphology for cognitively unimpaired individuals. We aimed to capitalize on the study of a large number of cognitively unimpaired late middle aged and older adults with two, one and no APOE-e4 alleles (Caselli et al., 2004) to characterize the ability of our automated hippocampal morphometry algorithm to distinguish between these three levels of genetic risk for AD and demonstrate its superiority to a commonly used hippocampal volume measurement. With the cross-sectional structural MR imaging data and APOE-e4 genotypes of 117 cognitively unimpaired individuals, we set out to test this hypothesis by computing bilateral hippocampal morphometries and analyzing morphometric differences related to the APOE-e4 dose effect.

## 2 Materials and methods

### 2.1 Database

Since January 1, 1994, cognitively normal residents of Maricopa County aged 21 years and older have been recruited through local media ads into the Arizona APOE cohort, a longitudinal study of cognitive aging (Caselli et al., 2004). Demographic, family, and medical history data is obtained on each individual undergoing APOE genotyping, and their identity is coded by a study assistant. All individuals give their written, informed consent—approved by the Institutional Review Boards of all participating institutions—and agree to have the results of the APOE test withheld from them as a precondition to their participation in this study. Genetic determination of APOE allelic status is performed using a polymerase chain reaction (PCR) based assay (Hixson and Vernier, 1990).

The recruitment strategy for the Arizona APOE cohort involves matching two e4 carriers and two e4 non-carriers by age, gender, and education. Screening tests include a medical history, neurologic examination, the Folstein Mini–Mental Status Exam (MMSE), the Auditory Verbal Learning Test Long-Term-Memory Scale (AVLT-LTM), the Hamilton Depression Rating Scale (Ham-D), the Functional Activities Questionnaire (FAQ), the Instrumental Activities of Daily Living (IADL) scale, and the Structured Psychiatric Interview for DSM-III-R. The study excludes subjects with potentially confounding medical, neurological, or psychiatric problems (such as prior stroke, traumatic brain injury, memory, or other cognitive impairment, parkinsonism, major depression, or substance abuse). No subject included in the study has the published criteria for MCI (Petersen et al., 2001), AD (Braun et al., 2008), any other form of dementia (Petersen et al., 2001), or major depressive disorder (Kok et al., 2009). Subjects fulfilling these requirements receive an extensive standardized battery of neuropsychological tests for one to two years. On the basis of age, gender, APOE genotype, educational background, and cognitive performance, we randomly selected subjects of each genotype with matched demographic information for brain scanning and deriving a normative imaging sample representative of the larger cohort. This subset of individuals formed the basis for this study. All subjects were scanned on the same GE 3Tesla scanner. A high-resolution T1 magnetization-prepared spoiled gradient (SPGR) scan was obtained in the sagittal plane using the same parameters as in the ADNI study. All scans were checked for movement and other quality measures prior to post-processing.

In this study, we sought to focus on the influence APOE-e4, and due to the protective influence of APOE-e2 against AD, APOE-e2 carriers were excluded (but will be the focus of a future analysis). The selected 117 cognitively healthy subjects aged between 50 and 85 years (mean = 57.4, SD = 6.3) were separated into three subgroups according to the number of e4 alleles: 44 NC, 36 HT, and 37 HM. Among them, 34 NCs, 31 HTs, and 32 HMs reported a first-degree family history of probable AD; 5 NCs, 5 HTs and 2 HMs had no first-degree family history; 5 NCs and 2 HMs did not report the first-degree family history. A T1-weighted pulse sequence (radiofrequency-SPGR recall acquisition in the steady state, repetition time = 33 msec, echo time = 5 msec, alpha = 30°, number of excitations = 1, field-of-view = 24 cm, imaging matrix = 256 × 92, slice thickness = 1.5 mm, scan time = 13:36 min) was used to acquire 124 contiguous horizontal MRI slices with in-plane voxel dimensions of 0.94 × 1.25 mm. T1-weighted MRIs were examined visually to ensure their freedom from artifacts, lacunar infarcts, and other clinically significant brain abnormalities. The database acquisition for this study was under the guidelines approved by the human subjects committees at Banner Good Samaritan Medical Center and the Mayo Clinic.

### 2.2 Processing pipeline

**Fig. 1:**
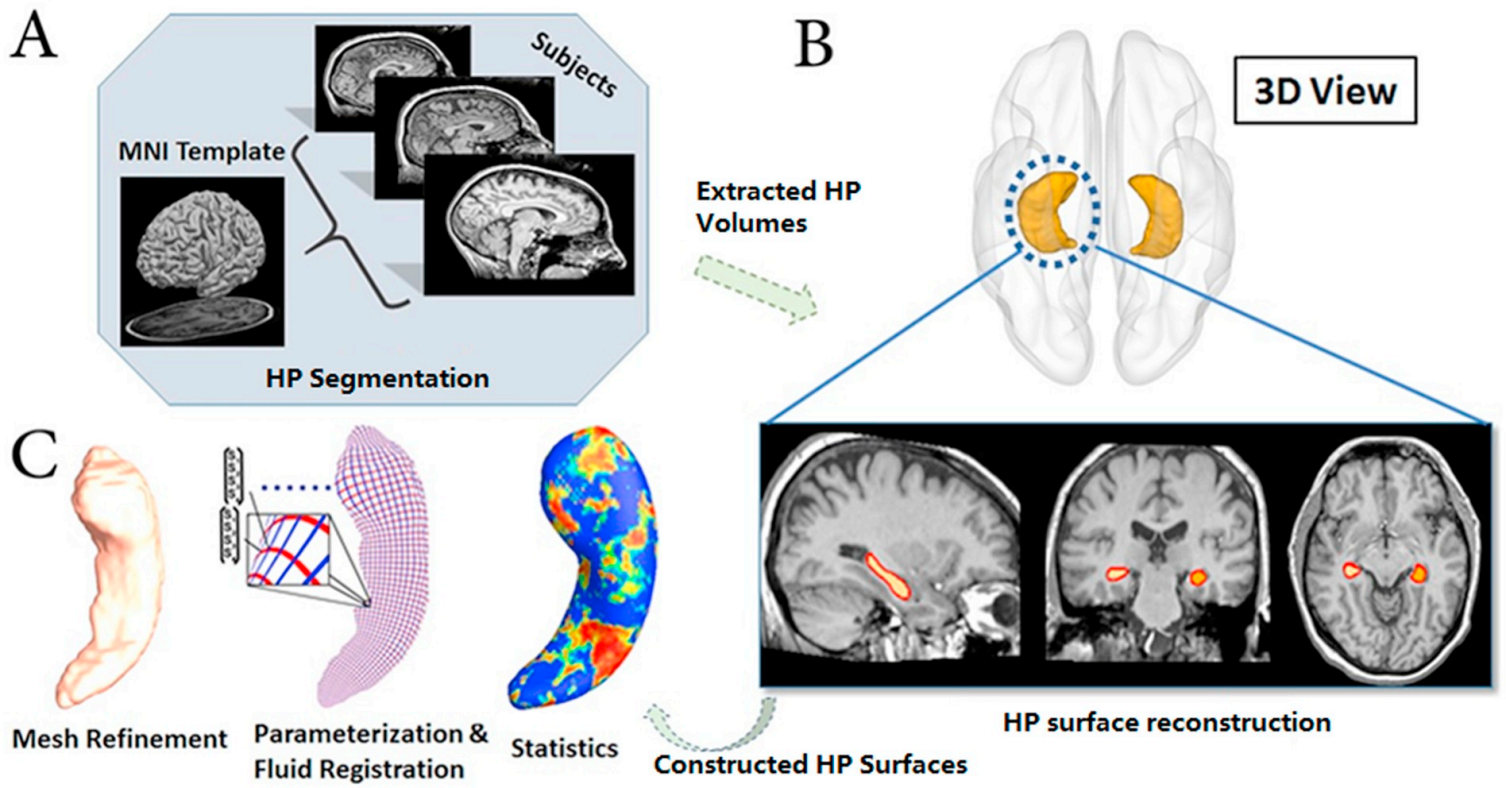
The pipeline of hippocampal morphometry analysis: (A) Hippocampi were automatically registered and segmented with FIRST (FMRIB's integrated registration and segmentation tool) (Patenaude et al., 2011); (B) Triangular surface meshes were constructed based on the extracted hippocampal volumes; (C) Each hippocampal surface mesh was parameterized on the refined triangular mesh and then registered to a common template for morphometric features extraction. Eventually, the group differences of hippocampal morphometry were statistically analyzed between different APOE genotype groups.

Our previous work (Shi et al., 2014) proposed a novel hippocampal surface morphometry method, which performed well for studying APOE-e4 dose-dependent effects on the hippocampal deformations of non-demented groups. The current work adopted a similar strategy to study APOE-e4 effects on the hippocampal morphometry of cognitively unimpaired people, as shown in Fig. 1. With FIRST in the FMRIB Software Library (FSL), hippocampal structures were segmented in the MNI152 standard space (Patenaude et al., 2011; Paquette et al., 2017) (see Fig. 1A). Surface meshes were constructed based on the hippocampal segmentations with the marching cubes algorithm (Lorensen and Cline, 1987) and a topology-preserving level set method (Han et al., 2003) (see Fig. 1B). Using the holomorphic flow segmentation method (Wang et al., 2007), each hippocampal surface was parameterized with refined triangular meshes, and the parameterized surfaces were then registered to a common rectangular grid template using the surface fluid registration algorithm (see Fig. 1C). To evaluate the deformation, the hippocampal morphometric features were extracted by concatenating the mTBM and the radial distance features. Eventually, by applying Hotelling's *T*^*2*^ test with a permutation test, differences of hippocampal morphometry among the APOE-e4 genotype groups were studied.

#### 2.2.1 Hippocampus segmentation and hippocampal surface reconstruction

The automated hippocampus segmentations from individual T1-weighted MR images were conducted using FIRST with default parameters (Patenaude et al., 2011) in FSL. The segmentation results of the bilateral hippocampi were manually inspected by projecting the results back to the original structural brain images, so that segment errors could be corrected. There were 2 subjects with significant segmentation errors, i.e., a small cluster of outlier voxels, in the binary mask of hippocampus. We manually remove those voxels and then reconstruct the hippocampus surfaces. Then, the left and right hippocampal surfaces were modeled with a topology-preserving level set method (Han et al., 2003). Based on the voxel-wise binary surfaces, the marching cubes algorithm (Lorensen and Cline, 1987) was applied to generate the triangular surface meshes. To refine the generated mesh and reduce the noise and obtuse angles, we further smoothed surfaces using progressive meshes (Hoppe, 1996) and loop subdivision (Loop, 1987) that down-sampled the mesh to a consistent number of vertexes (15,000 vertexes for each side of the hippocampus).

#### 2.2.2 Conformal parameterization-based hippocampal surface registration

Generally, morphometry analysis requires defining a common geometrical structure on surfaces so that all surfaces can be parameterized to a canonical space for the following surface registration and group differences estimation, e.g. (Thompson et al., 2004b; Fischl, 2012). To generate a planar surface conformal parameterization for a closed hippocampal surface, the topological optimization algorithm (Wang et al., 2012; Shi et al., 2013) was applied to automatically generate two cuts on each hippocampal surface, converting it to a genus zero surface with two open boundaries. The location of the two cuts were at the front and back of the hippocampal surface representing its anterior junction with the amygdala, and its posterior limit as it turned into the white matter of the fornix. They were biologically valid and consistent landmarks across subjects. These two landmark curves were automatically determined by searching along the first principle direction of geometric moments of hippocampal surfaces (Wang et al., 2011) and were manually inspected for quality control. Next, the holomorphic 1-form was computed. It induced a conformal grid which demonstrated the angle preserving property on the tube-like hippocampal surfaces (Wang et al., 2007). For more technical details about the holomorphic1-form, refer to our prior work (Wang et al., 2007; Wang et al., 2011).

In the next step, we registered each individual parameterized hippocampal surface to a common template surface. With conformal representations (Shi et al., 2013), a 3D hippocampus surface can be realized as a 2D image so that general image registration algorithms can be applied. In this study, we carried out a surface fluid registration algorithm (Bro-Nielsen and Gramkow, 1996; D'Agostino et al., 2003) and added an inverse-consistent surface registration framework to increase robustness (Shi et al., 2013). The obtained surface registration was guaranteed to be diffeomorphic and thus independent of the order of source and target images. The surface registration process is genetic and does not need any training data. More detailed algorithm description can be found in (Shi et al., 2013).

#### 2.2.3 Hippocampal morphometric feature extraction

After aligning the hippocampal surfaces for all participants, we computed their vertex-wise features with MMS, which consisted of two measures: mTBM and radial distance, mTBM, a 3 x 1vector, was computed as the matrix logarithm of the deformation tensor, which was generally referred to as the “Log-Euclidean metric” (Arsigny et al., 2006). Suppose *φ* : *S*_1_ → *S*_2_ is a registration map from surface *S*_1_ to a template surface *S*_2_, in the grid surface, the derivative map *dφ* is approximated by the linear map from one face [*v*_1_, *v*_2_, *v*_3_] to another face [*w*_1_, *w*_2_, *w*_3_], the planar coordinates of the vertices *v*_*i*_, *w*_*i*_ are denoted by the same symbol *v*_*i*_, *w*_*i*_ (Shi et al., 2015). Then the Jacobian matrix of *dφ* can be computed as (Wang et al., 2009):

$$J=\left[w_{3}-w_{1},w_{2}-w_{1}\right]{\left[v_{3}-v_{1},v_{3}-v_{1}\right]}^{-1}$$

TBM is defined as$\;\sqrt{\det J}$, where det*J* is the determinant of Jacobian matrix. mTBM can be expressed as$\;\log\sqrt{JJ^{T}}$. The mTBM has been widely used in brain structural research and outperforms TBM results (Leporé et al., 2008; Wang et al., 2010; Shi et al., 2013). It is sensitive to deformations such as rotation, dilation, and shear along the surface tangent direction, therefore the mTBM can effectively capture hippocampal structural alterations (e.g. atrophy and enlargement) in tensor fields.

The other measure, radial distance (Pizer et al., 1999), was suitable for tube-like shape analysis due to the computation of distance from a surface point to its medial core (i.e., the corresponding point in the centerline of the tube). Radial distance has been applied in several subcortical studies (Gerig et al., 2001; Morra et al., 2009; Thompson et al., 2004a, Thompson et al., 2004b) and served as an ideal complement to mTBM statistics for a comprehensive description of the hippocampal structural changes.

Finally, MMS for each vertex in the individual hippocampal surface was formed as a 4 × 1 vector by combining the mTBM and the radial distance. In other words, if there were *W* vertexes in the template surface and all subjects' hippocampal surfaces had been registered to it, the left or right hippocampal morphometry for each subject could be presented as a *W* × 4 feature matrix.

#### 2.2.4 Statistical group differences of hippocampal morphometry

Statistical hippocampal morphometry differences were analyzed between different genotype groups with Hotelling's *T*^*2*^ test (Cao and Worsley, 1999; Hotelling, 1992), vertex by vertex. That is, for each vertex, the group mean difference of two genotype groups of 4-dimensional vectors, S_i_(i = 1, 2,  … , N_S_) and T_j_(j = 1, 2,  … , N_T_), was measured using Mahalanobis distance M, defined as:

$$M=\frac{N_{S}N_{T}}{N_{S}+N_{T}}{\left(\bar{S}-\bar{T}\right)}^{T}\sum^{-1}\left(\bar{S}-\bar{T}\right)$$

N_S_ and N_T_ were the subject amounts of the two groups, $\bar{S}$ and $\bar{T}$ were the means of two group morphometry vectors, and ∑ was the combined covariance matrix of the two group morphometry vectors (Leporé et al., 2008; Wang et al., 2010; Wang et al., 2011).

After calculating the ground truth group difference of two groups at each vertex, we ran a permutation test with 10,000 repeats. For each repetition, all samples were randomly pooled into two groups, and the group Mahalanobis distance was computed. A probability (uncorrected *p*-value) on each vertex was defined as the ratio of the number of random permutation values greater than the ground truth group difference value to the total permutation times. Across all the vertices, the hippocampal morphometric group differences were shown in the form of a *p*-map. After that, a *p* feature was defined as the number of vertices with uncorrected *p*-values lower than the threshold (*p* < .05). The *p* feature was regarded as the real effect in the true experiment. By comparing the real *p* feature to the 10,000 *p* features derived from the random tests, we obtained a ratio describing what fraction of the time an effect of similar or greater magnitude to the real effect that occurred in the random assignments. This ratio, the *overall (corrected) significance*, was the chance of the observed pattern occurring by accident, which provided a global significance level of the map, and we accepted the permutation *p*-map if the ratio was <0.05.

Furthermore, the direction (atrophy or expansion) of group differences were analyzed at each surface point, we mapped the determinant of Jacobian matrix (det*J*) at each significant surface point *k* of subject group 1 and group 2 in a ratio map according to the following formula:

$$R^{k}=\frac{\sum_{i}^{N_{1}}\det J_{1i}^{k}}{\sum_{j}^{N_{2}}\det J_{2j}^{k}}\;\frac{N_{2}}{N_{1}}$$

where *J*_1*i*_^*k*^ and *J*_2*j*_^*k*^ are the Jacobian matrices for *i*th subject in group1 and *j*th in group2, *N*_1_ and *N*_2_ are the number of subjects in each group. Under the significant level (*p* < .05), *R*^*k*^ > 1 indicates that group2 has an atrophy at a given surface point contrast to group1, *R*^*k*^ < 1 indicates that group2 has an expansion at a given surface point contrast to group1 (Yao et al., 2018).

## 3 Results

### 3.1 Study samples

Demographic and clinical data were compared using a one-way analysis of variance, while data related to gender factor was analyzed using a chi-squared test (Crivello et al., 2010). The statistical results are summarized in Table 1, demonstrating that the demographic characteristics of the three groups are matched.

**Table 1:** Demographic characteristic statistics between genotype groups.

	NC	HT	HM	Inferential statistics
Sample size	44	36	37	
Age	58.6 (7.2)	57.2 (3.8)	58.4 (6.8)	F = 0.6; *p* = .56
Education	15.8 (2.3)	15.8 (2.4)	16.1 (2.1)	F = 0.2; *p* = .81
Male/female	15/29	11/25	9/28	χ^2^ = 0.9; *p* = .63
MMSE score	29.7 (0.6)	29.9 (0.4)	29.6 (0.7)	F = 1.7; *p* = .19
AVLT-LTM	8.75 (2.95)	9.86 (2.86)	10.03 (3.07)	F = 2.3; *p* = .1

Values are mean and (standard deviation) when applicable. NC: no-carriers; HT: heterozygotes; HM: homozygotes.

### 3.2 Hippocampal volume estimates of three levels of APOE-e4 genotype groups

Since volumetric measure is a widely-used index to reveal hippocampal atrophies related with AD pathology (Cohen et al., 2001; den Heijer et al., 2010; Moon et al., 2018), we first calculated the hippocampal volumetric measures on this dataset. Similar to prior approaches that used hippocampal volume for AD diagnosis, e.g. (Pennanen et al., 2004; Sandstrom et al., 2006; Chupin et al., 2007; Chupin et al., 2009; Pardoe et al., 2009), the hippocampal volumes were computed on the smoothed hippocampal structures after they were linearly registered to the MNI imaging space (Patenaude et al., 2011; Shi et al., 2013). Table 2 shows the volume means (standard deviations) of three genotype groups.

**Table 2:** Volumes of bilateral hippocampi of three genotypes on cognitively unimpaired cohort.

	NC	HT	HM
LH_volume	4705.27 (542.16)	4679.57 (489.08)	4736.04 (449.99)
RH_volume	4836.15 (440.74)	4844.11 (474.21)	4844.37 (486.29)

Values are mean and (standard deviation) when applicable, LH: left hippocampus; RH: right hippocampus; NC: no-carriers; HT: heterozygotes; HM: homozygotes.

### 3.3 Hippocampal morphometric differences of the contrast APOE-e4 carriers vs. NC in cognitively unimpaired individuals

**Fig. 2:**
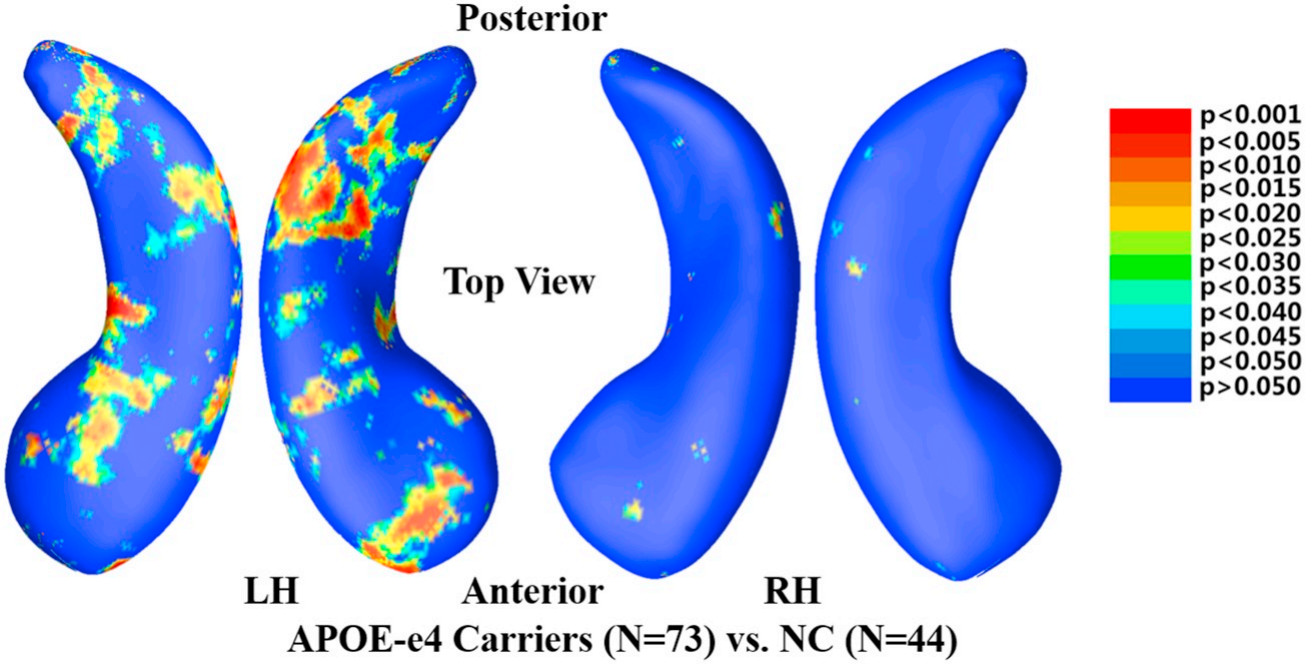
Group hippocampal shape differences between APOE-e4 allele (e3/e4 and e4/e4, N = 73) and NC (e3/e3, N = 44) on the cognitively unimpaired individuals, at the nominal 0.05 level, uncorrected. The overall significance of LH with permutation test was *p* < .02. However, the overall significance of RH with permutation test was not significant (*p* > .05). LH: left hippocampus; RH: right hippocampus; NC: non-carriers.

Many studies pooled HT and HM into the APOE-e4 carrier category and observed significant hippocampal volume losses for the carriers using longitudinal analysis (Moffat et al., 2000; Reiter et al., 2017) in cognitively unimpaired individuals. However, most cross-sectional studies failed to observe significant volume losses for the carriers (Mondadori et al., 2007; Burggren et al., 2008; Protas et al., 2013). We estimated the hippocampal deformations with cross-sectional analysis and expected to observe significant hippocampal deformations between APOE-e4 carriers and non-carriers. The group hippocampal morphometric comparisons were conducted between 44 NC and 73 e4 carriers (36 HT and 37 HM). Fig. 2 shows the *p*-maps of group differences on the LH and right hippocampus (RH). Non-blue colors show vertices with statistical differences at the nominal 0.05 level, uncorrected for multiple comparisons. We found significant morphometric differences on the LH (*p* < .02, corrected); however, no overall significances were observed on the RH (*p* > .05, corrected).

### 3.4 Hippocampal morphometric differences of the contrast HM vs. HT in cognitively unimpaired individuals

**Fig. 3:**
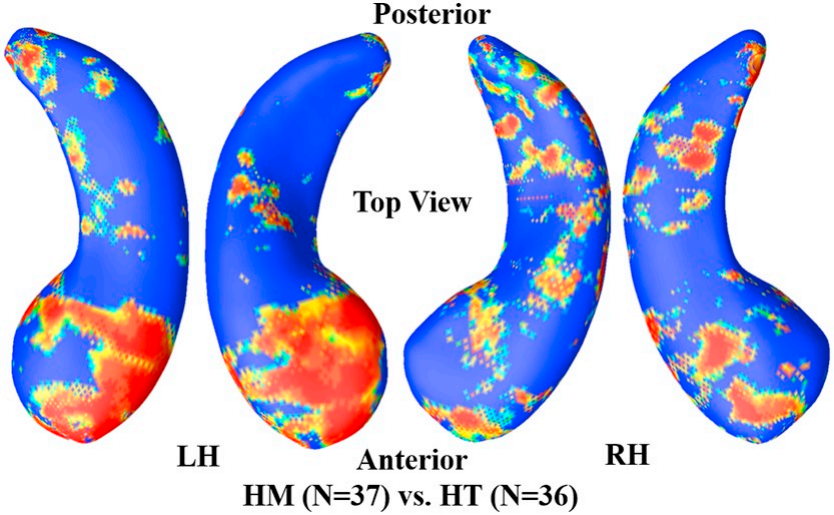
Group hippocampal shape differences between HM (e4/e4, *N* = 37) and HT (e3/e4, *N* = 36) in the cognitively unimpaired cohort, at the nominal 0.05 level, uncorrected. The overall significance of LH with permutation test was *p* < .01. The overall significance of RH with permutation test was p < .01. LH: left hippocampus; RH: right hippocampus; HM: homozygotes; HT: heterozygotes.

Among cognitively unimpaired individuals, many studies failed to identify hippocampal volume differences between HT and HM (Lemaitre et al., 2005; Crivello et al., 2010). In our work, to further study whether there are significant hippocampal morphometry differences between HT and HM in cognitively unimpaired individuals, group hippocampal morphometric comparisons were conducted between 36 HT and 37 HM individuals. Fig. 3 shows the contrast between the two groups as a statistical *p*-map of the LH and the RH, bilateral hippocampal atrophies of HT were significantly different from HM (*p* < .01, corrected).

### 3.5 Hippocampal morphometric differences of the contrast between carriers with different APOE-e4 dose and NC in cognitively unimpaired individuals

APOE-e4 dose effects on the hippocampal morphometry of the non-demented cohort have been reported in our prior work (Shi et al., 2014; Li et al., 2016). However, its effects on cognitively unimpaired people are still unclear. Therefore, in the cognitively unimpaired cohort, we studied group hippocampal morphometric differences between NC and HT/HM, hypothesizing that hippocampal morphometry could reveal the APOE-e4 dose effects. That is, hippocampal morphometry should reveal more pronounced differences between HM vs. NC than between HT vs. NC.

**Fig. 4:**
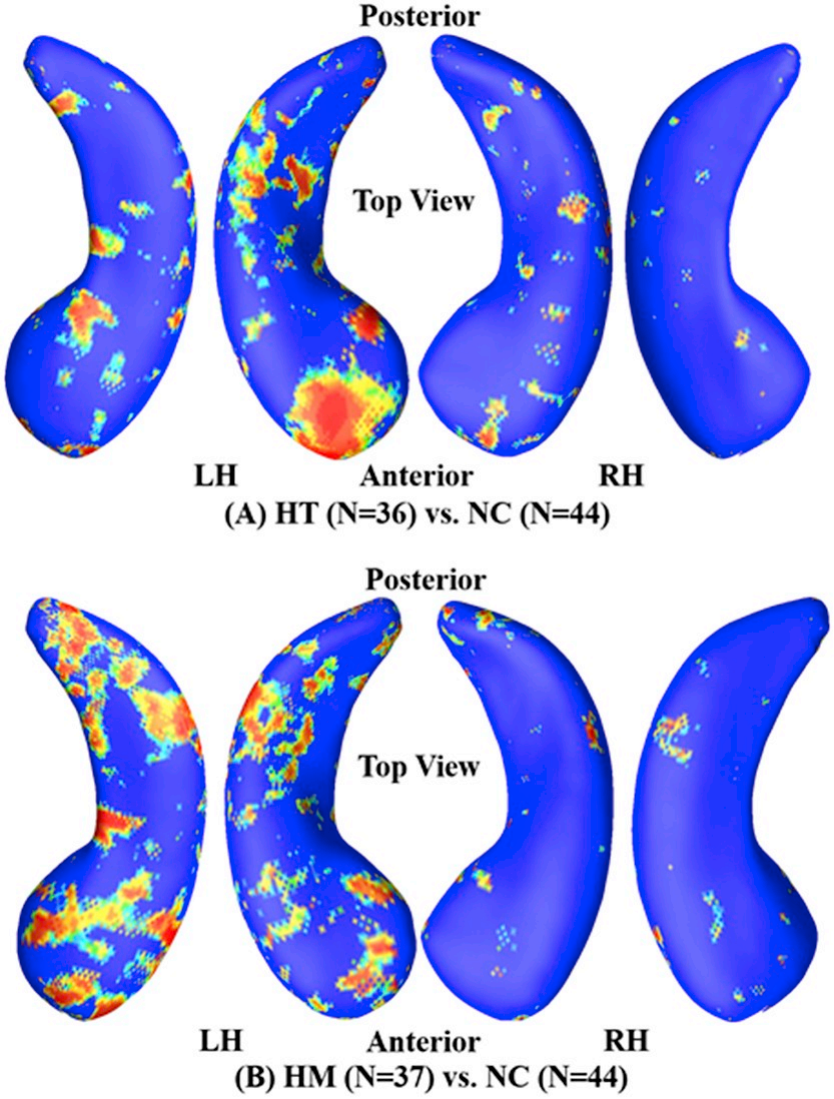
Group hippocampal shape differences between HT (e3/e4, *N* = 36) /HM (e4/e4, *N* = 37) and NC (e3/e3, *N* = 44) on the cognitively unimpaired cohort, at the nominal 0.05 level, uncorrected. (A) Between NC and HE, the overall significances after multiple comparisons with permutation test were P_LH_ = 0.0296 and P_RH_ = 0.3579. (B) Between NC and HM, the overall significances after multiple comparisons with permutation test were P_LH_ = 0.0105 and P_RH_ = 0.1886. LH: left hippocampus; RH: right hippocampus; HT: heterozygotes; HM: homozygotes; NC: no-carriers.

The statistical *p*-maps for the cognitively unimpaired cohort are shown in Fig. 4. Fig. 4A shows the comparison of HT vs. NC among the cognitively unimpaired individuals. We found significant atrophic differences on the LH (*p* < .03) but no overall significant differences on the RH (*p* > .05). As shown in Fig. 4B, we found more extensively significant (*p* < .02) differences on the LH surface for the HM vs. NC comparison of cognitively unimpaired individuals than those in Fig. 4A. The surface of RH does not present overall significances (*p* > .05).

### 3.6 Directional deformations of the hippocampal morphometric contrasts

**Fig. 5:**
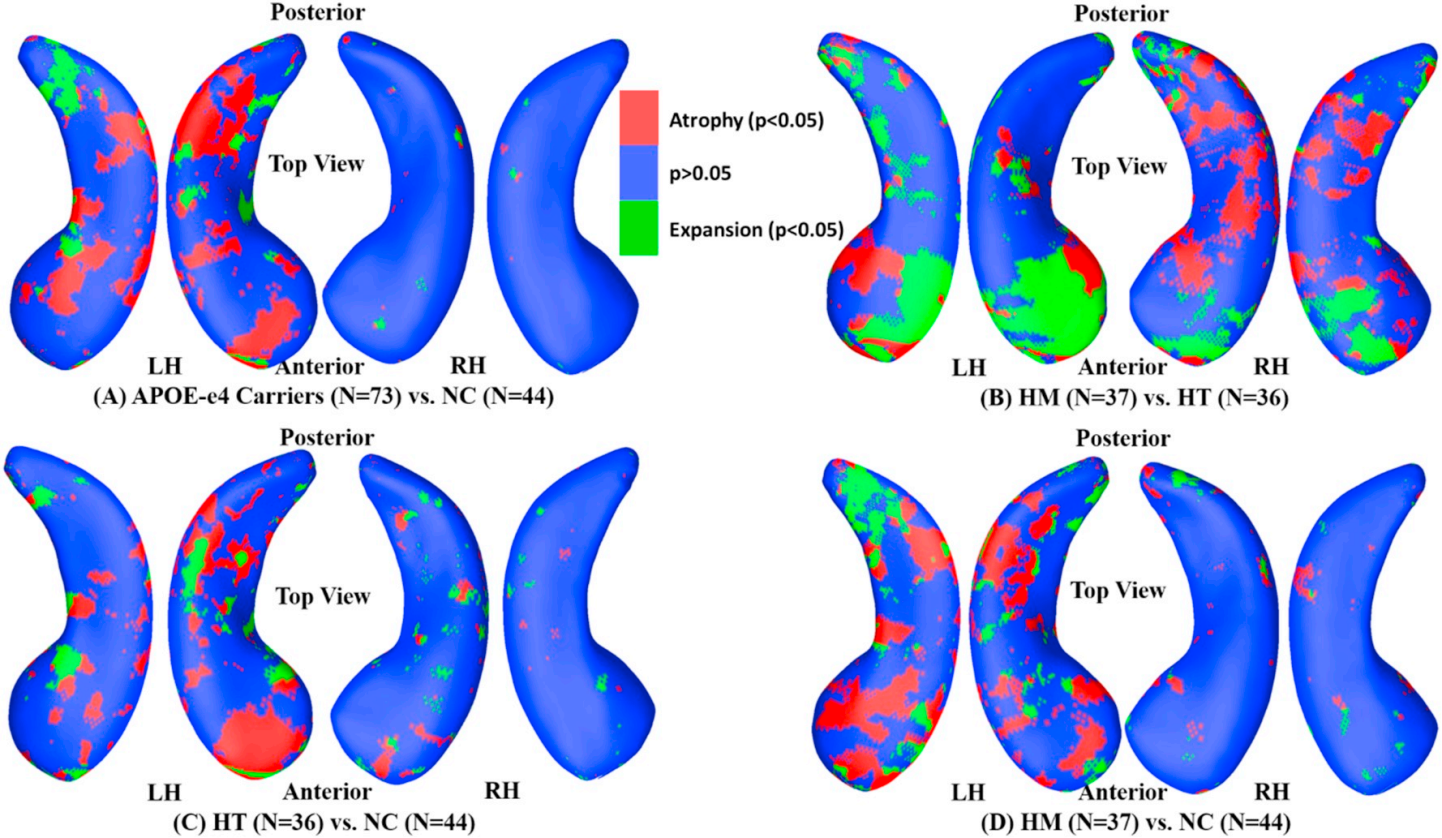
Illustrations of the directional deformations of the significant hippocampal regions of APOE-e4 carriers (e3/e4 and e4/e4, *N* = 73) compared to NC (e3/e3, *N* = 44; A), of HM (e4/e4, *N* = 37) compared to HT (e3/e4, *N* = 36; B), of HT (e3/e4, N = 36) compared to NC (e3/e3, N = 44; C), and of HM (e4/e4, N = 37) compared to NC (e3/e3, N = 44; D) in the cognitively unimpaired subjects. Red and green colors show vertices with significant atrophies and expansions of APOE-e4 carriers compared to NC at the nominal 0.05 level, uncorrected for multiple comparisons. LH: left hippocampus; RH: right hippocampus; NC: non-carriers.

Additionally, we analyzed the directional deformations of these group contrasts. As shown in Fig. 5, blue color represents insignificant regions in the comparison, red and green colors show areas with significant atrophies and expansions at the nominal 0.05 level, uncorrected for multiple comparisons. Fig. 5A shows that the LH of APOE-e4 carriers has larger atrophic areas, the RH of APOE-e4 carriers generally has no significant deformations contrast to NC. Fig. 5B shows bilateral hippocampal atrophies and expansions of HM compared to HT. As shown in Fig. 5C, LH of HT mainly shows significant atrophies, while RH of HT shows little deformations compared to NC. Fig. 5D shows significant LH deformations of HM are mainly atrophic compared to NC, while RH of HM shows little deformations relative to NC.

Regarding the expansion areas, one explanation could be that although there is always hippocampal atrophy, related either to normal aging (Kaye et al., 2005; Henneman et al., 2009) or AD progression (Reiman et al., 1998; Jack Jr. et al., 2003), there may be different atrophy patterns associated with NC, HT and HM subjects. As a result, those areas with more dramatic atrophy with HT are shown as expansion in the contrast between HT and HM (Fig. 5B). It may be also true for three other group contrasts (Fig. 5A, B and C).

### 3.7 Cumulative distribution analysis of the hippocampal morphometric comparisons

**Fig. 6:**
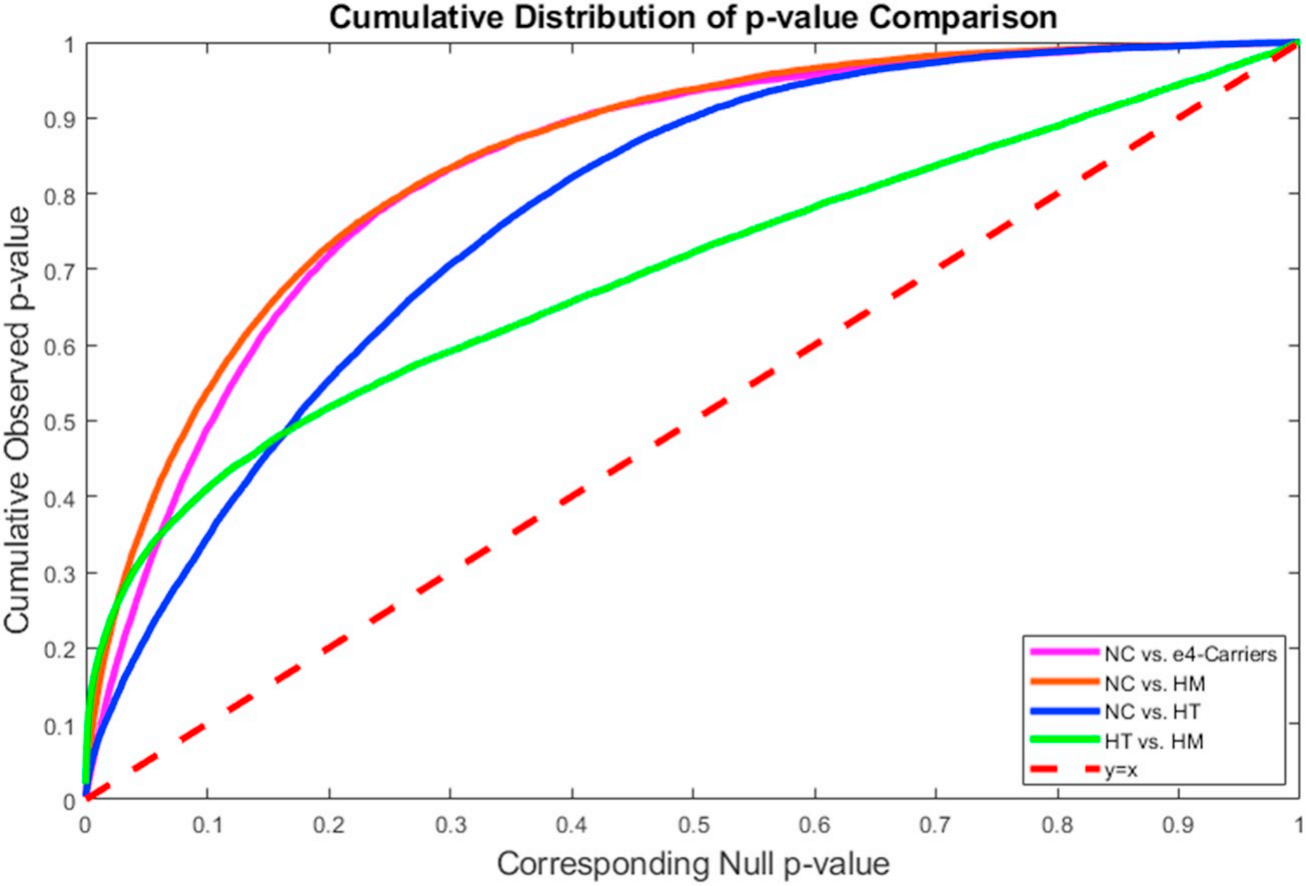
Cumulative distribution functions of the *p*-values from the LH morphometric comparisons of NC vs. e4-carriers, NC vs. HM, NC vs. HT and HT vs. HM, plotted against the expected *p*-values under the null hypothesis of no group differences among the comparisons. In false discovery rate methods, any cumulative distribution plot that rises steeply is a sign of a significant signal being detected, with curves that rise faster denoting higher effect sizes. The steep rise of the cumulative plot relative to *p*-values that would be expected by chance can be used to compare the detection sensitivity of different statistics derived from the same data. The deviations of the statistics from the null distribution generally increased from NC vs. HT to NC vs. HM in the cognitively unimpaired individuals study, suggesting that the APOE-e4 allele dose may be associated with faster atrophy of the LH. LH: left hippocampus; NC: no-carriers; HT: heterozygotes; HM: homozygotes.

Except for the significant morphometric differences between the RH surfaces of HT and HM, no other pair-wise significant differences were observed on the RH. To further validate the APOE-e4 allele dose effects on the LH atrophies, in Fig. 6, the cumulative distribution functions (CDF) of the *p*-values observed for the contrasts among cognitively unimpaired individuals with two, one and no APOE-e4 alleles are plotted against the corresponding *p*-value that would be expected, under the null hypothesis of no group difference, as used in our prior work (Wang et al., 2010; Wang et al., 2011; Shi et al., 2013; Wang et al., 2013; Shi et al., 2014). For null distributions, the CDF of *p*-values is expected to fall approximately along the dotted line; large deviations from that curve are associated with significant signal, and greater effect sizes are represented by larger deviation (the theory of false discovery rates gives formulate for thresholds that control false positives at a known rate).

As shown in Fig. 6, we note that the curve deviation of the statistics from the null distribution generally increases with the number of APOE-e4 alleles, curve deviations of the comparison of NC vs. HM (brown line) typically are larger than the comparison of NC vs. HT (blue line), which suggested a clear APOE-e4 dose effect. Additionally, large curve deviations of the comparison APOE-e4 carriers vs. NC means there are significant LH morphometric differences between carriers and NC, which is consistent with prior studies (Burggren et al., 2008; Reiter et al., 2017). The curve deviations of the comparison HT vs. HM are also larger than the null hypothesis distribution, it means that there are significant LH morphometric differences between HT and HM in cognitively unimpaired individuals, which was not reported by any existing studies, e.g. (Burggren et al., 2008; Mondadori et al., 2007; Protas et al., 2013).

## 4 Discussion

By analyzing cross-sectional structural MR images, the present study has revealed that the APOE-e4 allele has a dose-dependent impact on the left hippocampal morphology of 117 cognitively unimpaired individuals. Significant hippocampal deformations were observed in the comparisons of different genotype groups. The results validated our hypothesis that surface-based hippocampal morphometry analysis could identify the dose effect of the APOE-e4 allele on cognitively unimpaired individuals. To compare the performance of the proposed morphometry measure with the volume measure, we also made genotype volume contrasts on the same dataset. Table 3 summarizes the *p* values of the morphometry and volume measures in distinguishing different genotype groups. None of the hippocampal volume comparisons were significant (*p* > .05), but our hippocampal morphometry comparisons showed profound APOE-e4 dose effects. To our knowledge, this is the first study to use the surface-based hippocampal morphometry approach to successfully distinguish cognitively unimpaired persons with two, one, and no APOE-e4 alleles, i.e., deformations of LH_HM_ > deformations of LH_HT_ > deformations of LH_NC_. In particular, this work is among the first work to report HT having significant LH morphometric differences compared to HM/NC in cognitively unimpaired individuals.

**Table 3:** Statistics of the hippocampal volume and morphometry differences between genotype groups on cognitively unimpaired cohort.

	E4 carriers vs. NC	HT vs. NC	HM vs. NC	HT vs. HM
LH_volume	0.4877	0.4131	0.3921	0.3046
LH_morphometry	0.0165⁎	0.0296⁎	0.0105⁎	0.0095⁎
RH_volume	0.4636	0.4691	0.4683	0.4991
RH_morphometry	0.5992	0.3579	0.1886	0.0114⁎

Values are statistical *p* values. LH: left hippocampus; RH: right hippo-campus; NC: no-carriers; HT: heterozygotes; HM: homozygotes.

⁎ *p* values < 0.05.

### 4.1 Hippocampal morphometric differences of APOE-e4 carriers vs. NC

Medial temporal lobe structures are the earliest affected brain regions in people with AD (Chapleau et al., 2016) but APOE-e4 dose effects on general hippocampal morphology of cognitively unimpaired individuals remain unclear. Studies of cognitively unimpaired individuals often pool HT and HM into one APOE-e4 carrier category and report, after longitudinal analysis, significant hippocampal grey matter volume losses in carriers when compared to NC (Reiter et al., 2017). However, most of cross-sectional studies did not find significant group volume differences in the cognitively unimpaired cohorts (Mondadori et al., 2007; Burggren et al., 2008; Protas et al., 2013), a finding which was confirmed in this work, as shown in Table 3. The results of this study indicated there were significant left hippocampal morphometric differences (*p* < .05) between cognitively unimpaired APOE-e4 carriers and NC. These results are supported by previous longitudinal studies (Moffat et al., 2000; Reiter et al., 2017) and demonstrate that our hippocampal surface morphometry may outperform the volume measures in distinguishing cognitively unimpaired APOE-e4 carriers from NC using a cross-sectional analysis strategy.

### 4.2 Hippocampal morphometric differences of NC vs. HT vs. HM

Significant hippocampal morphometric differences (*p* < .05) were observed in the comparisons of cognitively unimpaired HM vs. HT/NC. These findings mirror studies that found significant hippocampal grey matter decreased in cognitively unimpaired HM compared to HT/NC (Lemaitre et al., 2005; Crivello et al., 2010). Here we observed significant morphometric differences on the LH. Our results are supported by (Morra et al., 2009), which conducted similar experiments with an ADNI baseline dataset (*N* = 490) and found significant differences only on the LH of healthy controls. Similarly, Southana et al. applied functional MRI in healthy subjects with known APOE-e4 genotypes, and found reduced neural activity in left hippocampal subregions of APOE-e4 carriers (Suthana et al., 2010). The study of (Foley et al., 2017) indicated that the onset of left hippocampus atrophy was earlier than the right hippocampus in 272 young healthy adults (average age: 24.8 years, SD 6.9) with higher polygenic risk scores. The study (Moon et al., 2018) suggested that left hippocampus had a faster volume reduction than the right side in the APOE-e4 carriers of 50 MCI patients aged 55–63 years. Another APOE-e4 study (Pievani et al., 2011) with manually segmented hippocampal surfaces is also consistent with our findings.

The key finding of this work is our observation of significant hippocampal morphometric differences between NC and HT, while such significant differences are not identified by the volumetric measure, as shown in Table 3. The studies of (Lemaitre et al., 2005; Crivello et al., 2010) applied the hippocampal volume measures to estimate the APOE-e4 dose effect on the hippocampal structure, but failed to find significant hippocampal deformations in HT compared to NC. Therefore, they concluded APOE-e4 effects on cortical atrophy were limited to HM, and the dose effect of APOE-e4 on brain structures was largely delayed in time. However, with the help of the hippocampal surface morphometry measure, our work demonstrated the APOE dose effect on hippocampal structure could be observed in an earlier stage.

### 4.3 Our findings for preclinical AD research

Prior work indicated that asymptomatic AD starts from left hippocampal atrophy decades before memory decline (Shi et al., 2009; Foley et al., 2017; Moon et al., 2018). Meanwhile, studies suggested that APOE-e4 is the major genetic risk for AD (Corder et al., 1993; Saunders et al., 1993). The APOE-e4 carriers have shown to be less efficient in extracellular fibrillary amyloid β (Aβ) plaques clearance than other isoforms, and the Aβ peptide may further affect hippocampal morphometry in an early stage (Liu et al., 2013; Cacciaglia et al., 2018). The atrophic process of hippocampal volume size met the left-less-than-right asymmetry pattern in cognitively unimpaired subjects (Shi et al., 2009). Our current findings, supported by some prior work (Lemaitre et al., 2005; Shi et al., 2014; Li et al., 2016), identified that APOE-e4 carriers of cognitively unimpaired subjects had significant left hippocampal atrophy. Our results supported that APOE-e4 is the major genetic risk for late-onset AD. Our work also indicated that the proposed surface-based hippocampal morphometry is capable to detect hippocampal deformations related with APOE-e4 before dementia symptom appearance and may serve as a valuable preclinical AD imaging biomarker.

### 4.4 Deformation directionality analysis

Similar to what we did in our prior work (Lao et al., 2016; Yao et al., 2018), we plotted the deformation directionality of the four studied group differences in Fig. 5. The contrast of HM vs. NC had more atrophic regions (see Fig. 5D) and stronger effect (see Fig. 6) than the contrast of HT vs. NC, so hippocampal morphometry may reveal the APOE-e4 dose effects on the LH deformations: HM > HT > NC. However, HT and HM had different hippocampal atrophic patterns relative to NC. On LH, HM had larger atrophic regions than HT (see Fig. 5C, D), while in some subregions, HT subjects express deeper LH atrophic than HM (see Fig. 5B). Considering that there were no significant LH volume differences in the contrast of HM vs. HT (see Table 3), with the directionality map (see Fig. 5B) we may infer that the LH atrophic regions of HT and LH atrophic regions of HM may cancel each other out in the form of LH volume differences. This observation probably helps explain why there were no significant LH volume differences between HM and HT in the early stage (Moffat et al., 2000; Lemaitre et al., 2005; Crivello et al., 2010). It also demonstrated that our surface-based subregional analysis may have stronger statistical power to detect some subtle brain morphometry differences years before the possible onset of dementia.

Likewise, we hypothesize that there are also different atrophy patterns associated with APOE-e4 on the right hippocampus. There is no statistically significant different areas in the contrasts of HT vs. NC and HM vs. NC. However, when comparing the right hippocampal morphometry between HT and HM, the different atrophy patterns made the overall significances after multiple comparisons (*p* < .012). Although further investigations (especially with larger datasets) are warranted to validate our hypothesis, the significance discovered between HT and HM on the right hippocampus does not necessarily contradict our observed APOE-e4 dose effects.

### 4.5 Subject ages of the studied cohort

The average age across of our subjects was under 60 years, and although relatively young is appropriate for preclinical analysis of individuals at high risk for future AD (APOE-e4 homozygotes) whose mean age of onset is typically between the late 60's and mid 70's. Younger patients less frequently have mixed degenerative pathologies at autopsy than older patients and so our findings may not be perfectly applicable to much older cohorts. Still the current work is complementary to our prior discoveries in ADNI, a relatively older cohort (Shi et al., 2014; Li et al., 2016).

### 4.6 Limitation and future work

Despite the promising results obtained by applying our automated surface-based morphometry system to MRIs of cognitively unimpaired APOE-e4 carriers and non-carriers, there were two important caveats. First, this work was based on cross-sectional MRI analysis and compared the hippocampal morphometries of cognitively unimpaired HM/HT/NC without considering the temporal trends of the hippocampal deformation. In future, we will conduct longitudinal analyses of cognitively unimpaired individuals as well. Second, it would be useful to check and compare the prediction power on subsequent memory decline with our surface multivariate statistics versus hippocampal volumes. For example, our prior work (Caselli et al., 2009) reported the longitudinal Auditory Verbal Learning Test Long-Term-Memory score (AVLT-LTM) as a sensitive measure for detecting accelerated memory decline in subjects with the APOE-e4 allele who were over the age of 50 years. In the present work, we only had cross-sectional AVLT-LTM scales of 117 subjects, and we did not find significant genetic group differences (see Table 1). In our future work, we will study the correlations between longitudinal surface-based hippocampal morphometry deformations and AVLT-LTM declines in each genetic groups. It will help add new insights into a better understanding of the surface-based hippocampal morphometry and their effectiveness as a potential preclinical AD biomarker.

## 5 Conclusion

This work proposed to apply a novel surface-based hippocampal morphometry measure to study the APOE-e4 dose effects on a cognitively unimpaired cohort. Results showed that the proposed approach encoded a great deal of information that may be inaccessible or overlooked by volume measures. This work found additive APOE-e4 effects on the left hippocampal morphometry of cognitively unimpaired individuals. The results, combined with our previous findings in the ADNI database (Shi et al., 2014; Li et al., 2016), support prior reports that the APOE-e4 genotype is associated with accelerated brain deformations along with disease progression, and that these differences can be mapped to morphological changes in subsections of the hippocampal surfaces. The work also demonstrated that our surface-based morphometry analysis may serve as a useful brain imaging marker to study AD induced brain morphometry changes in preclinical AD stage.
